# Presence of IL-17 in synovial fluid identifies a potential inflammatory osteoarthritic phenotype

**DOI:** 10.1371/journal.pone.0175109

**Published:** 2017-04-11

**Authors:** Sarah J. B. Snelling, Sylvette Bas, Gabor J. Puskas, Stephanie G. Dakin, Domizio Suva, Axel Finckh, Cem Gabay, Pierre Hoffmeyer, Andrew J. Carr, Anne Lübbeke

**Affiliations:** 1 Nuffield Department of Orthopaedics, Rheumatology and Musculoskeletal Sciences, University of Oxford, Oxford, United Kingdom; 2 Division of Rheumatology, Geneva University Hospitals, Geneva, Switzerland; 3 Division of Orthopaedic Surgery and Traumatology, Geneva University Hospitals, Geneva, Switzerland; University of Sao Paulo, BRAZIL

## Abstract

**Purpose:**

Osteoarthritis (OA) is a common and heterogeneous arthritic disorder. Patients suffer pain and their joints are characterized by articular cartilage loss and osteophyte formation. Risk factors for OA include age and obesity with inflammation identified as a key mediator of disease pathogenesis. Interleukin-17A (IL-17) is a pro-inflammatory cytokine that has been implicated in inflammatory diseases such as rheumatoid arthritis. IL-17 can upregulate expression of inflammatory cytokines and adipocytokines. The aim of this study was to evaluate IL-17 levels in the synovial fluid of patients with end-stage knee and hip OA in relation to inflammation- and pain-related cytokines and adipocytokines in synovial fluid and serum, and clinical and radiographic disease parameters.

**Methods:**

This is a cross-sectional study of 152 patients undergoing total hip and knee arthroplasty for OA. IL-17, IL-6, leptin, adiponectin, visfatin, resistin, C-C Motif Chemokine Ligand 2 (CCL2), C-C Motif Chemokine Ligand 7 (CCL7) and nerve growth factor (NGF) protein levels were measured in synovial fluid and serum using enzyme-linked immunosorbent assay (ELISA). Baseline characteristics included age, sex, body mass index, co-morbidities, pain and function, and radiographic analyses (OA features, K&L grade, minimal joint space width).

**Results:**

14 patients (9.2%) had detectable IL-17 in synovial fluid. These patients had significantly higher median concentrations of IL-6, leptin, resistin, CCL7 and NGF. Osteophytes, sclerosis and minimum joint space width were significantly reduced in patients with detectable IL-17 in synovial fluid. No differences were found in pain, function and comorbidities. IL-17 concentrations in synovial fluid and serum were moderately correlated (r = 0.482).

**Conclusion:**

The presence of IL-17 in the synovial fluid therefore identifies a substantial subset of primary end-stage OA patients with distinct biological and clinical features. Stratification of patients on the basis of IL-17 may identify those responsive to therapeutic targeting.

## Introduction

Osteoarthritis (OA) is the most common of the arthritic diseases causing pain and disability for sufferers. The disease is characterized by loss of articular cartilage and formation of osteophytes with synovial inflammation (synovitis) present in a significant proportion of patients. [1] Risk factors for this complex, multifactorial disease include female sex, age and obesity. Long-regarded as a non-inflammatory, degenerative disease, recent research consistently identifies a role of inflammation in driving both OA initiation and progression.[1, 2] A further shift in the OA disease paradigm is its consideration not as a single disease but instead as a series of different joint disorders that converge upon the common characteristic of articular cartilage loss in the absence of other pathology such as rheumatoid arthritis.

Individuals with OA show heterogeneity in clinical features including pain, speed of disease progression, bony changes (osteophyte formation, sclerosis of subchondral bone), synovitis and functional scores. This hinders prognostic, diagnostic and therapeutic advances, and treatments are limited to analgesia and arthroplasty. Successful development and application of interventions will rely upon a personalized medicine strategy enabled by disease stratification. Given the complexity of OA it is unlikely that a single cytokine drives disease progression nor identifies a disease subtype. Instead a cytokine network may act in concert in driving OA pathogenesis.[3] Studying serum or synovial fluid (SF) biomarkers alongside clinical and radiographic characteristics is one strategy to improve resolution and stratification into targetable OA phenotypes. Pro-inflammatory mediators such as interleukin-6 (IL-6) and tumour necrosis factor alpha (TNF) and adipocytokines such as leptin and resistin are detected in OA SF and serum and have been shown to correlate with disease progression.[4–6] The synovium is rich in immune cell populations and synovial fibroblasts and these are a potential source of inflammatory cytokines in OA. Adipocytokines and inflammatory cytokines are also produced by adipocytes and inflammatory cells, which are present in both joint-resident and systemic adipose tissue.[6, 7] The presence of inflammatory cytokines and adipokines in OA SF is in agreement with both inflammatory and metabolic or obesity-related risk factors as key drivers of OA.

Interleukin-17 (IL-17) is a pro-inflammatory cytokine that is strongly implicated in autoimmune disorders including rheumatoid arthritis, psoriatic arthritis and ankylosing spondylitis. Secreted by cells types including T-helper-17 (Th17), mast and myeloid cells, IL-17 promotes production and release of pro-inflammatory cytokines including IL-6 from chondrocytes and synovial fibroblasts.[8] IL-17 can also drive synovial fibroblast and inflammatory cell survival and increases expression of the monocyte chemoattractants C-C Motif Chemokine Ligand 2 (CCL2) and C-C Motif Chemokine Ligand 7 (CCL7).[9] The interplay of IL-6 and IL-17 has been suggested as crucial in the pathogenesis of chronic inflammatory diseases, with IL-6 inducing Th17 cell differentiation and thus IL-17 production independent of TNF and IL-1β [10, 11] In OA numerous studies have highlighted IL-6 as a biomarker that can drive cartilage degradation however any interaction between IL-17 and IL-6 in OA has not been well studied.[12, 13] IL-17 has also been shown to disrupt extracellular matrix (ECM) homeostasis both independently and via synergy with other pro-inflammatory cytokines[14, 15] or adipocytokines[16]. Alongside cartilage loss and consequent joint space narrowing, a key feature of OA is pain, and IL-17 has also been identified as a pain sensitizer in rodent models of arthritis.[17]

It is compelling that previous studies have shown that IL-17 can drive cartilage destruction, bone remodeling and pain sensitization as well as increase the expression of inflammatory cytokines and adipocytokines. However most studies of IL-17 have focused on autoimmune diseases rather than OA. There is a particular paucity of work assessing IL-17 levels in relation to OA-relevant cytokines, adipocytokines, and radiographic and clinical features. The aim of this work was to measure IL-17 levels in OA patients with end-stage hip and knee disease and assess its relationship with clinical and radiographic parameters of OA, and levels of adipokines, pro-inflammatory cytokines and pain-associated cytokines.

## Methods

This cross-sectional study was carried out at a large orthopaedic centre between January and December 2010. Patients recruited underwent total hip arthroplasty (THA) or total knee arthroplasty (TKA) following presentation with end-stage hip or knee OA. Patients with secondary OA due to trauma (known history of fracture or previous surgery) or known immune-mediated arthritis were excluded. The study was approved by the institutional review board of the Geneva University Hospitals and the patients' written informed consent was obtained (N° 09–215; NAC 09–072).

### Cytokine measurement

SF was collected during surgery via direct aspiration through the joint capsule immediately after skin incision. Blood samples (5 ml) were obtained in the morning during the pre-operative examination. All SF and blood samples were immediately centrifuged, aliquoted and stored at -80°C until use. Adipokines (leptin, resistin, adiponectin, visfatin) and cytokines (IL-17, IL-6, nerve growth factor (NGF), CCL2, CCL7) were measured in SF and serum using DuoSet enzyme-linked immunosorbent assay (ELISA, R&D Systems, Abingdon, UK). Minimum detectable cytokine concentrations for these assays were measured to be 1 pg/ml for IL-6, CCL7, NGF and IL-17, 31.2 pg/ml for leptin and resistin, 62.5 pg/ml for adiponectin and 15.6 pg/ml for CCL2. Both intra- and interassay coefficients of variation were < 10%. Visfatin concentrations were measured using a Phoenix ELISA kit (Phoenix Pharmaceuticals, Inc., Burlingame, CA, USA) according to the manufacturer. The estimated minimum detectable visfatin concentration was 2.2 ng/ml (interassay variation <15% and intraessay variation <10%).

### Clinical and radiographic parameters

Clinical parameters assessed preoperatively included age, sex, BMI, co-morbidities, smoking status, medication use, duration of symptoms, pain and function using visual analogue scale (VAS) and Western Ontario and McMaster Universities Arthritis Index (WOMAC) scores. General health (SF-12 mental and physical component score) was also assessed. Preoperative radiograph evaluation included OA features (osteophytes, sclerosis), Kellgren & Lawrence (K&L) grade, and minimal joint space width (JSW).

### Statistical analysis

Fisher’s exact test was used to compare categorical variables between those with and without detectable IL-17 levels in SF. For continuous variables the independent samples median test was employed. Correlations between IL-17 and adipocytokines and cytokines in SF and serum were evaluated using Spearman’s correlation coefficient. Assumption of normality was checked by plotting frequency distributions and using the Kolmogorov-Smirnow test.

## Results

152 patients were recruited to the study, 68 prior to THA and 84 prior to TKA. Mean age was 73 (±9) years, 64% were women.

**Fig 1:**
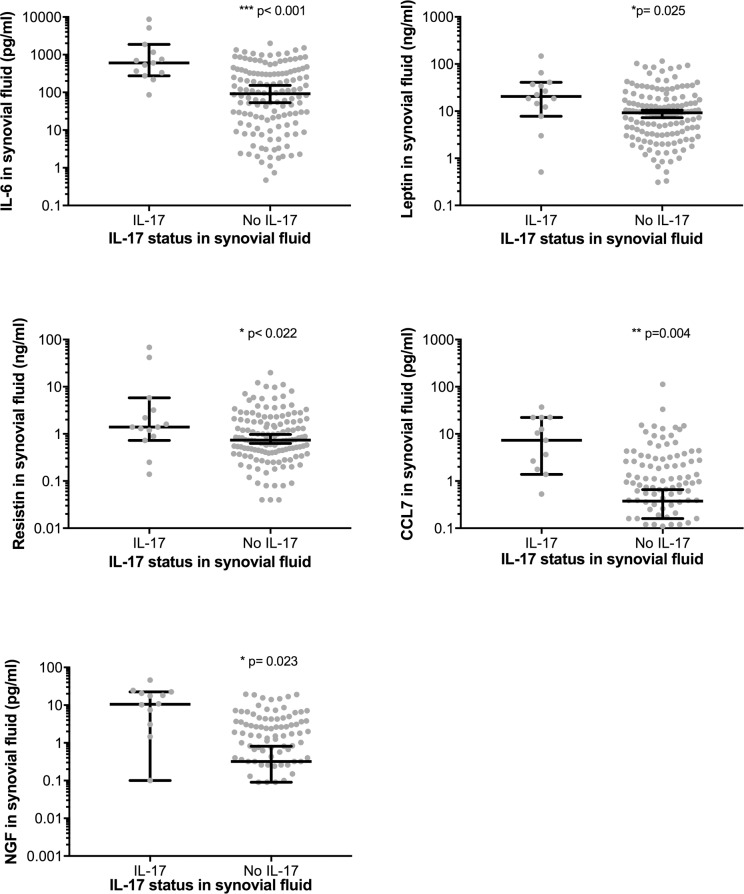
Synovial fluid concentrations of IL-6, Leptin, Resistin, CCL7 and NGF in patients with and without detectable synovial fluid IL-17 Error bars show median ± 95% confidence intervals, *p<0.05, **p<0.01, **p<0.001.

**Fig 2:**
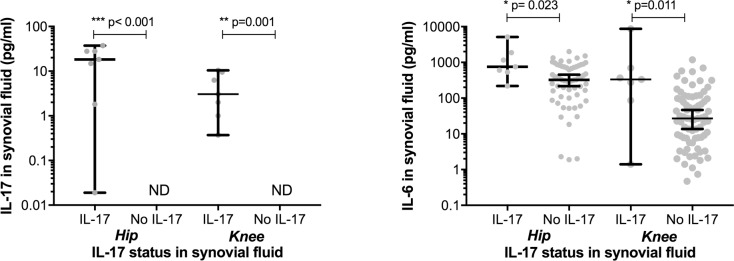
Synovial fluid concentrations of IL-17 and IL-6 in patients following stratification by hip or knee OA. Error bars show median ± 95% confidence intervals, *p<0.05, **p<0.01, **p<0.001, ND = not detectable.

In 138 patients no IL-17 was detected in the SF whilst 14 (9.6%) had detectable IL-17 (median concentration 7.9 pg/ml), (Table 1, Fig 1). Patients with detectable IL-17 had significantly increased IL-6 (568.5 pg/ml vs. 89.4 pg/ml, p = 0.005), leptin (20.5 ng/ml vs. 9.2 ng/ml, p = 0.025), resistin (1.4 ng/ml vs. 0.7 ng/ml, p = 0.022), CCL7 (8.9 pg/ml vs. 0.4 pg/ml, p = 0.004), and NGF (10.6 pg/ml vs. 0.3pg/ml, p = 0.023) SF concentrations. Adiponectin tended to be higher in the IL-17 group (0.5 μg/ml vs. 0.3 μg/ml, p = 0.092). Following stratification of patients by hip or knee OA both groups showed significantly increased IL-6 in those patients with detectable IL-17 (Fig 2). There was no significant difference in SF IL-17 or IL-6 concentration when hip and knee patients with detectable IL-17 were compared.

**Table 1:** Synovial fluid IL-17 levels in relation to synovial fluid and serum adipokines and cytokines.

**Synovial fluid concentration, median, IQR**	**n**	**Synovial fluid IL-17**	**n**	**Synovial fluid IL-17**	p-value*
		**Yes**		**No**	
IL-17 (pg/ml)	14	7.9 (1.6; 20.6)	138	0 (0; 0)	p<0.001
IL-6 (pg/ml)	14	568.5 (261.8; 1338.8)	138	89.4 (15.5; 333.8)	0.005
Leptin (ng/ml)	14	20.5 (11.2; 37.9)	138	9.2 (3.3; 18.8)	0.025
Adiponectin (μg/ml)	14	0.5 (0.3; 1.1)	138	0.3 (0.1; 0.6)	0.092
Visfatin (ng/ml)	14	8.3 (4.5; 13.7)	138	7.0 (4.3; 9.8)	1
Resistin (ng/ml)	14	1.4 (0.9; 3.9)	138	0.7 (0.4; 1.9)	0.022
CCL2 (pg/ml)	14	335.5 (153.3; 1095.3)	138	348.5 (187.8; 664.3)	1
CCL7 (pg/ml)	14	8.9 (1.7; 22.3)	138	0.4 (0; 2.1)	0.004
NGF (pg/ml)	14	10.6 (1.1; 21.2)	135	0.3 (0; 2.7)	0.023
**Serum concentration, median, IQR**					
IL-17 (pg/ml)	13	2.4 (0; 14.0)	115	0 (0; 0)	<0.001
IL-17 detectable in serum (%)	13	9/13 (69.2)	115	14/115 (12.2)	<0.001
IL-6 (pg/ml)	14	5.2 (1.1; 11.4)	117	1.0 (0.3; 5.9)	0.044
Leptin (ng/ml)	14	32.3 (15.7; 57.4)	117	15.1 (6.6; 41.1)	0.149
Adiponectin (μg/ml)	14	2.5 (1.6; 4.9)	117	2.5 (1.4; 4.9)	0.848
Visfatin (ng/ml)	14	11.8 (8.1; 16.2)	117	11.8 (8.9; 13.3)	0.801
Resistin (ng/ml)	14	6.8 (2.5; 10.5)	117	5.9 (3.6; 9.4)	0.754
CCL2 (pg/ml)	14	119.5 (74.3; 177.3)	116	169.5 (101.5; 234.5)	0.157
CCL7 (pg/ml)	14	3.2 (0.02; 16.9)	117	0 (0; 0.3)	0.01
NGF (pg/ml)	13	10.6 (1.1; 21.2)	116	0 (0; 0.6)	0.037
Serum leptin ≥15ng/ml in women/ ≥7.5ng/ml in men, (%)**	14	12 (85.7)	117	69 (59.0)	0.052

*p-values obtained with use of Fisher’s exact test for categorical variables and independent samples median test for continuous variables

**Leptin serum level cut-offs based on *Askari H et al*. *Fasting plasma leptin level is a surrogate measure of insulin sensitivity*. *J Clin Endocrinol Metab*. *2010*

Baseline and clinical characteristics and functional scores were not significantly different between patients with and without detectable SF IL-17 (Table 2). However radiographic characteristics were significantly altered (Table 3) with patients with detectable IL-17 having lower minimum JSW (median 0.2mm, interquartile range (IQR) 0–0.9 vs. median 1.0mm, IQR 0–2.0, p = 0.042), fewer osteophytes (61.5% vs. 94.7%, p = 0.001) and less sclerosis (46.2% vs. 83.3%, p = 0.005)

**Table 2:** Synovial fluid IL-17 levels in relation to baseline and clinical characteristics and patient-reported outcome measures.

**Baseline characteristics**	**n**	**Synovial fluid IL-17**	**n**	**Synovial fluid IL-17**	p-value*
		**Yes**		**No**	
Women (%)	14	12 (85.7)	138	85 (61.6)	0.086
Age, median, IQR	14	68 (63.8; 76.3)	138	74 (68.8; 80.0)	0.061
BMI, median, IQR	14	28.2 (25.2; 33.4)	138	27.3 (24.9; 29.4)	0.575
BMI ≥ 35 kg/m^2^ (%)	14	3 (21.4)	138	9 (6.5)	0.083
ASA classification 3/4 (%)*	14	2 (14.3)	138	32 (23.2)	0.737
Diabetes (%)	14	0	138	17 (12.3)	0.369
Hypertension (5)	14	9 (64.3)	138	86 (62.3)	1
Ever-smoker (%)	14	2 (14.3)	138	44 (32.1)	0.229
Statin user (%)	14	4 (28.6)	138	40 (29.0)	1
**Clinical characteristics**					
Hip osteoarthritis (%)	14	7 (50)	138	61 (44.2)	0.781
Contralateral joint symptomatic or replaced (%)	14	7 (50)	138	79 (57.2)	0.778
Duration of symptoms <1 year (%)**	7	3/7 (42.9)	35	6/35 (17.1)	0.155
Cortisone injection in months before surgery (%)	14	2 (14.3)	138	16 (11.6)	0.867
Synovitis intra-operative (%)***	7	7/7 (100)	70	50/70 (71.4)	0.18
**Patient-reported outcome measures**					
VAS pain, median, IQR	14	6 (4.8; 7.5)	138	6 (5; 8)	0.966
Moderate to very severe night pain (%)	14	13 (92.9)	127	93 (73.2)	0.189
WOMAC, median, IQR					
Pain	14	37.5 (23.8; 50)	127	40 (30; 50)	0.712
Function	14	42.9 (32.1; 57.1)	127	39.3 (25; 53.6)	0.167
SF-12, median, IQR					
Mental component score	14	40.8 (32; 52)	123	41.6 (34; 52)	0.8
Physical component score	14	31.4 (25.9; 32.8)	123	32.8 (27.4; 40.8)	0.167

*ASA = American Society of Anaesthesiology

**Information on duration of symptoms only available for 42 patients

***Synovitis absence or presence documented intra-operative only prior to total knee arthroplasty (n = 7 and n = 70)

**Table 3:** Synovial fluid IL-17 levels in relation to radiographic characteristics.

Radiographic characteristics	n	Synovial fluid IL-17	n	Synovial fluid IL-17	p-value*
		Yes		No	
K&L grade 3–4 (%)	13	11 (84.6)	132	121 (91.7)	0.329
MinJSW, mm, median, IQR	13	0.2 (0; 0.9)	132	1.0 (0; 2.0)	0.042
Osteophytes present (%)	13	8 (61.5)	132	125 (94.7)	0.001
Sclerosis present (%)	13	6 (46.2)	132	110 (83.3)	0.005

*p-values obtained with use of Fisher’s exact test for categorical variables and independent samples median test for continuous variables

**Fig 3:**
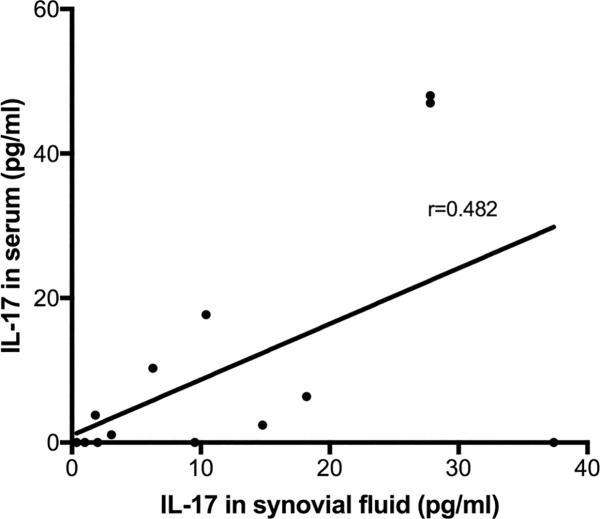
Correlation between synovial fluid and serum IL-17 concentrations in patients with detectable IL-17 in synovial fluid.

**Fig 4:**
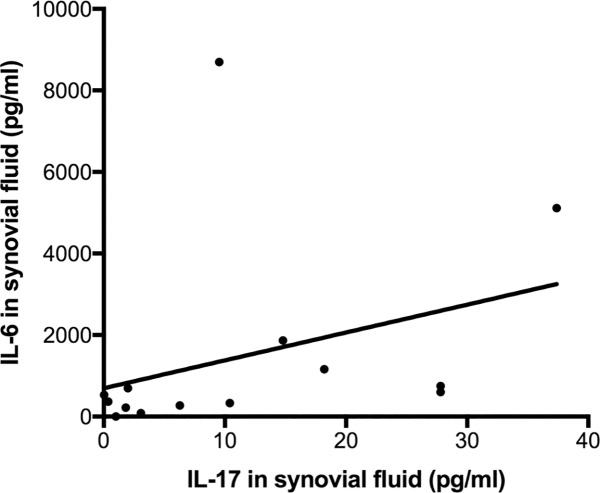
Correlation between synovial fluid IL-17 and IL-6 concentrations in patients with detectable IL-17 in synovial fluid.

Correlation between serum and SF IL-17 levels was moderate (r = 0.482, Fig 3). In line with SF adipokine and cytokine levels, serum IL-6, CCL7 and NGF were also significantly higher in patients with detectable IL-17 as compared to those without (Table 1). Synovial fluid IL-17 was significantly correlated with SF IL-6 (Fig 4), CCL7, NGF, Leptin and Resistin, and with serum IL-6, CCL7 and NGF (Table 4).

**Table 4:** Correlation between synovial fluid and serum IL-17 and the concentrations of adipokines and cytokines that show significant elevation in the cohort of patients with detectable synovial fluid IL-17.

	IL-6	CCL7	NGF	Leptin	Resistin
**Synovial fluid**					
**IL-17**	0.274**	0. 339**	0.307**	0.189*	0.161*
**Serum**					
**IL-17**	0.219*	0.534**	0.602**	0.129	0.006

Values represent Spearman's correlation coefficients

*Correlation is significant at the 0.05 level

**Correlation is significant at the 0.01 level

## Discussion

We show the presence of IL-17 in the SF of a subset of end-stage primary OA patients. This subset possesses a distinct radiographic, inflammatory cytokine and adipocytokine profile. This, to our knowledge, is the first study to identify an IL-17 driven stratification phenotype of OA.

Successful therapeutic intervention, mechanistic understanding and development of diagnostic and prognostic tools in OA require a clear definition of the phenotypes of disease. We propose that the presence of IL-17 in SF defines a distinct OA phenotype and may be targetable for therapeutic intervention. In the US OA is estimated to affect 33.7% of those aged over 65 with nearly one million hip and knee replacements performed in 2009 at an estimated cost of $42.3 million.[18, 19] Therefore the potential to identify and therapeutically target the 10% of OA patients corresponding to the IL-17-driven subset is of significant value. An aptamer against IL-17RA, the receptor for IL-17, prevented synovitis in a murine model of OA.[20] This further supports exploration of IL-17 as a therapeutic target. Clinical trials of IL-17-targeting therapeutics including secukinumab, a monoclonal antibody against IL-17, have shown promising but variable results in psoriatic arthritis and rheumatoid arthritis. This variability is partially attributed to heterogeneity in IL-17 levels between patients with the beneficial effects of anti-IL-17 therapeutics potentially seen in the patients with higher IL-17 levels.[21, 22] Therefore targeting the OA patient subset with detectable synovial fluid IL-17 may be of value in clinical trials of IL-17 based therapeutics in OA. Correlation of serum and synovial fluid IL-17 levels suggests potential of IL-17 as a non-invasive biomarker for disease stratification. This alongside the similar serum and SF adipokine and inflammatory cytokines fingerprint in those patients with detectable IL-17 suggests a systemic influence of IL-17 in these patients.

Disease stratification in OA is often based upon features including etiology, clinical presentation (sex, speed of progression, pain), comorbidities (obesity, metabolic syndrome), or nature of tissues and joints affected (polyarticular or monoarticular, atrophic or hypertrophic). In this study, we show that SF IL-17 associates with a phenotype of OA with reduced bony changes (osteophyte formation and sclerosis) but increased loss of JSW. Although statistical significance was not reached, patients with detectable SF IL-17 also tended to be younger, female and obese with faster progressing disease. The presence of IL-17 in SF may thus define a patient subset that would otherwise be classified as either atrophic or rapidly destructive; typically characterized by a lack of bony involvement but relative increase in synovitis and rate of progression.[23, 24]

In accordance with increased radiographic progression and reduced osteophytes and sclerosis, IL-17 promotes matrix metalloproteinase (MMP) production, enhances osteoclast and inhibits osteoblast activity[14, 25, 26]. The inflammatory phenotype of this patient subset is supported by previous reports correlating IL-17 with synovitis in rheumatoid arthritis and OA [27]. However, the presence of synovitis in a significant proportion of OA patients without detectable IL-17 suggests an independent inflammatory component to OA in these patients and further work is necessary to identify the cytokine networks responsible. The cytokine profile and synovitis in those patients with detectable IL-17 may be partially driven by IL17’s ability to recruit IL-17 producing inflammatory cells and promote synoviocyte survival[28]. As IL-17 induces release of IL-6 and CCL7 from synoviocytes, its presence in SF may drive the increased IL-6 and CCL7 levels observed in our patient cohort[29]. CCL7 is a monocyte chemoattractant and it may therefore act to initiate or maintain monocyte infiltration in OA-related synovitis.[30] Synergistic effects of IL-17 with these inflammatory cytokines may further enhance cartilage destruction and loss of JSW[14]. It is interesting to note that Th17 cells can transdifferentiate to regulatory T cells that promote resolution of inflammation and do not secrete IL-17.[31]. The presence of IL-17 in the SF and serum of a subset of end-stage OA patients in this study may suggest ongoing, unresolved inflammation -characterisitic of a chronic inflammatory disease. A recent genome wide expression analysis also highlighted IL-17 signaling as a key modulatory pathway of OA and suggested that IL-17 be used in *in vitro* OA models instead of the commonly used IL1β[27, 32]. IL-17 has previously been detected in the synovial fluid and serum of both controls and OA patients from a Chinese population.[33, 34] The incidence of hip OA is lower in Chinese populations[35, 36] and that of lateral knee OA significantly higher.[37] Furthermore the genetic associations underlying OA in Caucasian and Asian populations differ. [38] Therefore the differential detectability of IL-17 in serum and synovial fluid in our study can be attributed to the differences in both OA susceptibility and characteristics in Chinese compared to Caucasian populations.

Increased leptin and resistin define a distinct adipocytokine profile in n the patient subset with detectable IL-17. Adiposity in the absence of leptin signaling does not drive OA in animal studies.[39] The increased leptin and resistin in our study suggest an adiposity-related driver to disease in IL-17 patients, reflected in their trend towards an increased BMI. Leptin and resistin correlate with OA progression and severity, and induce MMP expression and release of inflammatory cytokines including IL-17[40]. Leptin also drives collagen-induced arthritis by enhancing Th17 cell generation.[41] In agreement with the cytokine profile of the IL-17 patient subset, IL-17 itself enhances adipocyte leptin, IL-6 and IL-8 secretion[42, 43]. IL-17 can also inhibit adipogenesis and impair obesity development in animal models and thus may be produced in response to increased adiposity or obesity in this patient subset.[42, 43]

This is an exploratory, cross-sectional study of end-stage OA and thus we cannot rule out a role of IL-17 in a larger subset of patients in early stage disease. Furthermore we cannot determine causality nor define the relative role or source of the IL-17 correlated cytokines in our patient cohort. IL-17 has been detected in the synovial tissue of OA patients, associated with macrophages, T-cells and in blood vessels but IL-17 levels were heterogeneous and not all will be secreted.[21] The limits of detection of ELISAs means that lower concentrations of IL-17 may be present within synovial fluid and serum. [22, 44] However future studies with more sensitive assays may identify a threshold IL-17 concentration above which the more inflammatory phenotype is present. The volume of SF aspirates also limited the number of cytokines that could be assessed by ELISA. The small patient sample size means patient stratification according to site of OA occurrence is underpowered. A longitudinal study in a larger patient cohort should explore SF and serum IL-17 levels in hip and knee OA patients alongside an enhanced panel of inflammatory cytokines and adipokines.

In summary, we show that IL-17 defines a specific subgroup of OA with reduced bony involvement and an increased inflammatory phenotype in the absence of metabolic syndrome. IL-17 may be therapeutically targetable in this subset.

## References

[1] Atukorala I, Kwoh CK, Guermazi A, Roemer FW, Boudreau RM, Hannon MJ, et al Synovitis in knee osteoarthritis: a precursor of disease? Annals of the rheumatic diseases. 2014. Epub 2014/12/10. 10.1136/annrheumdis-2014-205894 PMC4916836 25488799

[2] Kapoor M, Martel-Pelletier J, Lajeunesse D, Pelletier JP, Fahmi H. Role of proinflammatory cytokines in the pathophysiology of osteoarthritis. Nature reviews Rheumatology. 2011;7(1):33–42. Epub 2010/12/02. doi: 10.1038/nrrheum.2010.196 21119608 10.1038/nrrheum.2010.196

[3] Schett G, Elewaut D, McInnes IB, Dayer JM, Neurath MF. How cytokine networks fuel inflammation: Toward a cytokine-based disease taxonomy. Nat Med. 2013;19(7):822–4. doi: 10.1038/nm.3260 23836224 10.1038/nm.3260

[4] Karvonen-Gutierrez CA, Zheng H, Mancuso P, Harlow SD. Higher Leptin and Adiponectin Concentrations Predict Poorer Performance-based Physical Functioning in Midlife Women: the Michigan Study of Women's Health Across the Nation. The journals of gerontology Series A, Biological sciences and medical sciences. 2015. Epub 2015/08/26. 10.1093/gerona/glv123 PMC5014187 26302979

[5] Song YZ, Guan J, Wang HJ, Ma W, Li F, Xu F, et al Possible Involvement of Serum and Synovial Fluid Resistin in Knee Osteoarthritis: Cartilage Damage, Clinical, and Radiological Links. Journal of clinical laboratory analysis. 2015. Epub 2015/10/24. 10.1002/jcla.21876 PMC6807051 26494484

[6] Poonpet T, Honsawek S. Adipokines: Biomarkers for osteoarthritis? World journal of orthopedics. 2014;5(3):319–27. PubMed Central PMCID: PMCPMC4095025. doi: 10.5312/wjo.v5.i3.319 25035835 10.5312/wjo.v5.i3.319 PMC4095025

[7] Makki K, Froguel P, Wolowczuk I. Adipose tissue in obesity-related inflammation and insulin resistance: cells, cytokines, and chemokines. ISRN Inflamm. 2013;2013:139239 PubMed Central PMCID: PMCPMC3881510. doi: 10.1155/2013/139239 24455420 10.1155/2013/139239 PMC3881510

[8] Shahrara S, Pickens SR, Mandelin AM 2nd, Karpus WJ, Huang Q, Kolls JK, et al IL-17-mediated monocyte migration occurs partially through CC chemokine ligand 2/monocyte chemoattractant protein-1 induction. J Immunol. 2010;184(8):4479–87 PubMed Central PMCID: PMCPMC2858914. doi: 10.4049/jimmunol.0901942 20228199 10.4049/jimmunol.0901942 PMC2858914

[9] Benedetti G, Miossec P. Interleukin 17 contributes to the chronicity of inflammatory diseases such as rheumatoid arthritis. Eur J Immunol. 2014;44(2):339–47. doi: 10.1002/eji.201344184 24310226 10.1002/eji.201344184

[10] Kimura A, Naka T, Kishimoto T. IL-6-dependent and -independent pathways in the development of interleukin 17-producing T helper cells. Proc Natl Acad Sci U S A. 2007;104(29):12099–104. PubMed Central PMCID: PMCPMC1924582. doi: 10.1073/pnas.0705268104 17623780 10.1073/pnas.0705268104 PMC1924582

[11] Camporeale A, Poli V. IL-6, IL-17 and STAT3: a holy trinity in auto-immunity? Front Biosci (Landmark Ed). 2012;17:2306–26. 22652781 10.2741/4054

[12] Vida M, Gavito AL, Pavon FJ, Bautista D, Serrano A, Suarez J, et al Chronic administration of recombinant IL-6 upregulates lipogenic enzyme expression and aggravates high-fat-diet-induced steatosis in IL-6-deficient mice. Dis Model Mech. 2015;8(7):721–31. PubMed Central PMCID: PMCPMC4486858. doi: 10.1242/dmm.019166 26035386 10.1242/dmm.019166 PMC4486858

[13] Mabey T, Honsawek S. Cytokines as biochemical markers for knee osteoarthritis. World journal of orthopedics. 2015;6(1):95–105. PubMed Central PMCID: PMCPMC4303794. doi: 10.5312/wjo.v6.i1.95 25621214 10.5312/wjo.v6.i1.95 PMC4303794

[14] Koshy PJ, Henderson N, Logan C, Life PF, Cawston TE, Rowan AD. Interleukin 17 induces cartilage collagen breakdown: novel synergistic effects in combination with proinflammatory cytokines. Annals of the rheumatic diseases. 2002;61(8):704–13. Epub 2002/07/16. PubMed Central PMCID: PMC1754191. doi: 10.1136/ard.61.8.704 12117676 10.1136/ard.61.8.704 PMC1754191

[15] Chabaud M, Durand JM, Buchs N, Fossiez F, Page G, Frappart L, et al Human interleukin-17: A T cell-derived proinflammatory cytokine produced by the rheumatoid synovium. Arthritis and rheumatism. 1999;42(5):963–70. Epub 1999/05/14. doi: 10.1002/1529-0131(199905)42:5<963::AID-ANR15>3.0.CO;2-E 10323452 10.1002/1529-0131(199905)42:5<963::AID-ANR15>3.0.CO;2-E

[16] Wilke CM, Bishop K, Fox D, Zou W. Deciphering the role of Th17 cells in human disease. Trends in immunology. 2011;32(12):603–11. Epub 2011/10/01. PubMed Central PMCID: PMC3224806. doi: 10.1016/j.it.2011.08.003 21958759 10.1016/j.it.2011.08.003 PMC3224806

[17] Richter F, Natura G, Ebbinghaus M, von Banchet GS, Hensellek S, Konig C, et al Interleukin-17 sensitizes joint nociceptors to mechanical stimuli and contributes to arthritic pain through neuronal interleukin-17 receptors in rodents. Arthritis and rheumatism. 2012;64(12):4125–34. Epub 2012/11/30. doi: 10.1002/art.37695 23192794 10.1002/art.37695

[18] Murphy L, Helmick CG. The impact of osteoarthritis in the United States: a population-health perspective: A population-based review of the fourth most common cause of hospitalization in U.S. adults. Orthop Nurs. 2012;31(2):85–91. doi: 10.1097/NOR.0b013e31824fcd42 22446800 10.1097/NOR.0b013e31824fcd42

[19] Lawrence RC, Felson DT, Helmick CG, Arnold LM, Choi H, Deyo RA, et al Estimates of the prevalence of arthritis and other rheumatic conditions in the United States. Part II. Arthritis and rheumatism. 2008;58(1):26–35. PubMed Central PMCID: PMCPMC3266664. doi: 10.1002/art.23176 18163497 10.1002/art.23176 PMC3266664

[20] Chen L, Li DQ, Zhong J, Wu XL, Chen Q, Peng H, et al IL-17RA aptamer-mediated repression of IL-6 inhibits synovium inflammation in a murine model of osteoarthritis. Osteoarthritis and cartilage / OARS, Osteoarthritis Research Society. 2011;19(6):711–8. 10.1016/j.joca.2011.01.018 21310253

[21] van Baarsen LG, Lebre MC, van der Coelen D, Aarrass S, Tang MW, Ramwadhdoebe TH, et al Heterogeneous expression pattern of interleukin 17A (IL-17A), IL-17F and their receptors in synovium of rheumatoid arthritis, psoriatic arthritis and osteoarthritis: possible explanation for nonresponse to anti-IL-17 therapy? Arthritis research & therapy. 2014;16(4):426. Epub 2014/08/26. PubMed Central PMCID: PMC4292832. 25146432 10.1186/s13075-014-0426-z PMC4292832

[22] Mease PJ, McInnes IB, Kirkham B, Kavanaugh A, Rahman P, van der Heijde D, et al Secukinumab Inhibition of Interleukin-17A in Patients with Psoriatic Arthritis. N Engl J Med. 2015;373(14):1329–39. doi: 10.1056/NEJMoa1412679 26422723 10.1056/NEJMoa1412679

[23] Conrozier T, Ferrand F, Poole AR, Verret C, Mathieu P, Ionescu M, et al Differences in biomarkers of type II collagen in atrophic and hypertrophic osteoarthritis of the hip: implications for the differing pathobiologies. Osteoarthritis and cartilage / OARS, Osteoarthritis Research Society. 2007;15(4):462–7. Epub 2006/10/24. 10.1016/j.joca.2006.09.002 17055306

[24] Garnero P, Charni N, Juillet F, Conrozier T, Vignon E. Increased urinary type II collagen helical and C telopeptide levels are independently associated with a rapidly destructive hip osteoarthritis. Annals of the rheumatic diseases. 2006;65(12):1639–44. Epub 2006/03/30. PubMed Central PMCID: PMC1798449. doi: 10.1136/ard.2006.052621 16569684 10.1136/ard.2006.052621 PMC1798449

[25] Kim YG, Park JW, Lee JM, Suh JY, Lee JK, Chang BS, et al IL-17 inhibits osteoblast differentiation and bone regeneration in rat. Archives of oral biology. 2014;59(9):897–905. Epub 2014/06/08. doi: 10.1016/j.archoralbio.2014.05.009 24907519 10.1016/j.archoralbio.2014.05.009

[26] Kotake S, Udagawa N, Takahashi N, Matsuzaki K, Itoh K, Ishiyama S, et al IL-17 in synovial fluids from patients with rheumatoid arthritis is a potent stimulator of osteoclastogenesis. The Journal of clinical investigation. 1999;103(9):1345–52. Epub 1999/05/04. PubMed Central PMCID: PMC408356. doi: 10.1172/JCI5703 10225978 10.1172/JCI5703 PMC408356

[27] Deligne C, Casulli S, Pigenet A, Bougault C, Campillo-Gimenez L, Nourissat G, et al Differential expression of interleukin-17 and interleukin-22 in inflamed and non-inflamed synovium from osteoarthritis patients. Osteoarthritis and cartilage / OARS, Osteoarthritis Research Society. 2015;23(11):1843–52. Epub 2015/11/03. 10.1016/j.joca.2014.12.007 26521730

[28] Toh ML, Gonzales G, Koenders MI, Tournadre A, Boyle D, Lubberts E, et al Role of interleukin 17 in arthritis chronicity through survival of synoviocytes via regulation of synoviolin expression. PloS one. 2010;5(10):e13416 Epub 2010/10/27. PubMed Central PMCID: PMC2955522. doi: 10.1371/journal.pone.0013416 20976214 10.1371/journal.pone.0013416 PMC2955522

[29] Hwang SY, Kim JY, Kim KW, Park MK, Moon Y, Kim WU, et al IL-17 induces production of IL-6 and IL-8 in rheumatoid arthritis synovial fibroblasts via NF-kappaB- and PI3-kinase/Akt-dependent pathways. Arthritis research & therapy. 2004;6(2):R120–8. Epub 2004/04/03. PubMed Central PMCID: PMC400429. 15059275 10.1186/ar1038 PMC400429

[30] Shi C, Pamer EG. Monocyte recruitment during infection and inflammation. Nat Rev Immunol. 2011;11(11):762–74. doi: 10.1038/nri3070 21984070 10.1038/nri3070 PMC3947780

[31] Gagliani N, Vesely MC, Iseppon A, Brockmann L, Xu H, Palm NW, et al Th17 cells transdifferentiate into regulatory T cells during resolution of inflammation. Nature. 2015;523(7559):221–5. Epub 2015/04/30. PubMed Central PMCID: PMC4498984. doi: 10.1038/nature14452 25924064 10.1038/nature14452 PMC4498984

[32] Sandy JD, Chan DD, Trevino RL, Wimmer MA, Plaas A. Human genome-wide expression analysis reorients the study of inflammatory mediators and biomechanics in osteoarthritis. Osteoarthritis and cartilage / OARS, Osteoarthritis Research Society. 2015;23(11):1939–45. Epub 2015/11/03. PubMed Central PMCID: PMC4630670. 10.1016/j.joca.2015.03.027 PMC4630670 26521740

[33] Chen B, Deng Y, Tan Y, Qin J, Chen LB. Association between severity of knee osteoarthritis and serum and synovial fluid interleukin 17 concentrations. The Journal of international medical research. 2014;42(1):138–44. Epub 2013/12/10. doi: 10.1177/0300060513501751 24319050 10.1177/0300060513501751

[34] Liu Y, Peng H, Meng Z, Wei M. Correlation of IL-17 Level in Synovia and Severity of Knee Osteoarthritis. Med Sci Monit. 2015;21:1732–6. PubMed Central PMCID: PMCPMC4480114. doi: 10.12659/MSM.893771 26076201 10.12659/MSM.893771 PMC4480114

[35] Lau EM, Lin F, Lam D, Silman A, Croft P. Hip osteoarthritis and dysplasia in Chinese men. Annals of the rheumatic diseases. 1995;54(12):965–9. PubMed Central PMCID: PMCPMC1010061. 8546528 10.1136/ard.54.12.965 PMC1010061

[36] Kang X, Fransen M, Zhang Y, Li H, Ke Y, Lu M, et al The high prevalence of knee osteoarthritis in a rural Chinese population: the Wuchuan osteoarthritis study. Arthritis and rheumatism. 2009;61(5):641–7. PubMed Central PMCID: PMCPMC2758273. doi: 10.1002/art.24464 19405001 10.1002/art.24464 PMC2758273

[37] Nevitt MC, Xu L, Zhang Y, Lui LY, Yu W, Lane NE, et al Very low prevalence of hip osteoarthritis among Chinese elderly in Beijing, China, compared with whites in the United States: the Beijing osteoarthritis study. Arthritis and rheumatism. 2002;46(7):1773–9. doi: 10.1002/art.10332 12124860 10.1002/art.10332

[38] Dai J, Ikegawa S. Recent advances in association studies of osteoarthritis susceptibility genes. J Hum Genet. 2010;55(2):77–80. doi: 10.1038/jhg.2009.137 20075947 10.1038/jhg.2009.137

[39] Griffin TM, Huebner JL, Kraus VB, Guilak F. Extreme obesity due to impaired leptin signaling in mice does not cause knee osteoarthritis. Arthritis and rheumatism. 2009;60(10):2935–44. Epub 2009/10/01. PubMed Central PMCID: PMC2829313. doi: 10.1002/art.24854 19790050 10.1002/art.24854 PMC2829313

[40] Richter M, Trzeciak T, Owecki M, Pucher A, Kaczmarczyk J. The role of adipocytokines in the pathogenesis of knee joint osteoarthritis. International orthopaedics. 2015;39(6):1211–7. Epub 2015/02/27. doi: 10.1007/s00264-015-2707-9 25716111 10.1007/s00264-015-2707-9

[41] Deng J, Liu Y, Yang M, Wang S, Zhang M, Wang X, et al Leptin exacerbates collagen-induced arthritis via enhancement of Th17 cell response. Arthritis and rheumatism. 2012;64(11):3564–73. Epub 2012/07/27. doi: 10.1002/art.34637 22833425 10.1002/art.34637

[42] Noh M. Interleukin-17A increases leptin production in human bone marrow mesenchymal stem cells. Biochemical pharmacology. 2012;83(5):661–70. Epub 2011/12/27. doi: 10.1016/j.bcp.2011.12.010 22197587 10.1016/j.bcp.2011.12.010

[43] Shin JH, Shin DW, Noh M. Interleukin-17A inhibits adipocyte differentiation in human mesenchymal stem cells and regulates pro-inflammatory responses in adipocytes. Biochemical pharmacology. 2009;77(12):1835–44. Epub 2009/05/12. doi: 10.1016/j.bcp.2009.03.008 19428338 10.1016/j.bcp.2009.03.008

[44] Furst DE, Emery P. Rheumatoid arthritis pathophysiology: update on emerging cytokine and cytokine-associated cell targets. Rheumatology (Oxford). 2014;53(9):1560–9. PubMed Central PMCID: PMCPMC4135582. 24402580 10.1093/rheumatology/ket414 PMC4135582

